# Can really plasma interleukin-6 predict postoperative delirium among patients undergoing open repair surgery of acute type A aortic dissection?

**DOI:** 10.1186/s13019-022-01804-y

**Published:** 2022-03-27

**Authors:** Wen-He Yang, Fu-Shan Xue, Cheng-Wen Li

**Affiliations:** Department of Anesthesiology, Beijing Friendship Hospital, Capital Medical University, NO. 95 Yong-An Road, Xi-Cheng, Beijing, 100050 People’s Republic of China

**Keywords:** Postoperative delirium, Biomarker, Interleukin-6, Prediction, Acute aortic dissection

## Abstract

The letter to the editor was written in response to “Plasma interleukin-6 is a potential predictive biomarker for postoperative delirium (POD) among acute type a aortic dissection patients treated with open surgical repair”, which is recently published by Lv et al. (J Cardiothorac Surg 16(1):146, 2021). In this article, Lv et al. conclude that plasma IL-6 is a potential biomarker for prediction of POD. However, we note several issues in this study that would have made interpretation of their results questionable. Our main concerns include the use of a short POD assessment time, no providing the data of analgesics and sedatives used in the ICU, application of incorrect statistical methods when assessing predictive ability of plasma IL-6 for the development of POD, and incorrect interpretation for the area under the receiver operating characteristic curve. We believe that addressing these issues will improve the transparency of this study and help the interpretation of findings.

## To the Editor,

By a retrospective analysis of 221 consecutive patients undergoing open surgical repair combined with triple-branched stent graft implantation for type A acute aortic dissection (AAD), Lv et al. [1] assessed the performance of plasma Interleukin-6 (IL-6) as a potential biomarker to predict the development of postoperative delirium (POD). By the receiver operating characteristic (ROC) curve analysis, they conclude that plasma IL-6 is a potential biomarker for prediction of POD. As a small sample retrospective study, however, it unavoidably introduces a number of confounders. Other than the limitations described by the authors in the discussion, we wish to remind the readers of other issues in this study that would have biased their findings.

First, the data of this study was collected from March 2018 to January 2020, but only POD occurring within the first 3 postoperative days was diagnosed by the CAM-ICU method. In fact, the newest recommendations for nomenclature of cognitive change associated with anesthesia and surgery-2018 need that POD occurring in hospital up to 1 week after surgery or until discharge should be observed [2]. In fact, the incidence of POD in this study (13.2%, 26 of 196 patients) is significantly lower than those reported in available literatures (24.5–37.9%) [3–5]. As an inconsistent assessment time is one of main reasons that the incidence of POD varies among studies [7], we are concerned that use of a short assessment time of 3 days in this study would have underestimated the incidence of POD after open surgical repair of AAD. Furthermore, we believe that this study would have provided more accurate incidence of POD, if a prolonged assessment time was used, as performed in other studies assessing the occurrence and predictors of POD after open repair surgery of AAD [3, 4].

Second, mean times of ICU stay and ventilation support time were 4.97 days and 55 h, respectively. However, the readers were not provided with the details regarding management of analgesia and sedation in the ICU. The available evidence indicates that postoperative use of midazolam and morphine in the ICU are significantly associated with the developing POD after open surgical repair of AAD, especially when more than two types of analgesics and sedatives are used [3, 4]. We argue that these unknown factors would have further confounded the incidence of POD reported in this study.

Third, only use of the receiver operating characteristic (ROC) curve analysis to assess the predictive ability of plasma IL-6 for the development of POD is inappropriate. The authors should firstly determine the associations between plasma IL-6 and POD by univariate and multivariate logistic regression analyses. Because this study only included a small sample, moreover, the multivariate logistic regression model should include all previously reported risk factors associated with POD in the available literatures (including significant and nonsignificant variables following univariate analysis). Based on adjusted odds ratios and 95% confidence intervals of candidate variables obtained in the multivariate logistic regression analysis, independent contributions of significant factors to the development of POD can be determined. Only after a significant association between plasma IL6 and POD are demonstrated, the ROC curve analysis to assess the predictive performance of plasma IL-6 for POD can be carried out [6].

Fourth, the ROC curve analysis showed that the mean areas under the curves of preoperative and postoperative plasma IL6 levels for discrimination of POD only were 0.57–0.73, indicating that plasma IL6 levels only have a fair predictive value [7]. Other than the areas under the curve, optimal cutoff value, Youden index, sensitivity and specificity described in this study, after the ROC curve analysis, the authors should also provide the positive and negative predictive value of plasma IL6 for POD. According to all these data, the readers can determine how close is the predicted probability to the observed frequency.

## Data Availability

Not applicable.

## Abbreviations

AAD: Acute aortic dissection
POD: Postoperative delirium
ROC: Receiver operating characteristic curve
IL-6: Interleukin-6
